# Novel antioxidant protein target therapy to counter the prevalence and severity of SARS-CoV-2

**DOI:** 10.3389/fimmu.2023.1241313

**Published:** 2024-01-03

**Authors:** Priyajit Kaur, Akash Dey, Kartik Rawat, Sharmistha Dey

**Affiliations:** ^1^ Department of Biophysics, All India Institute of Medical Sciences, New Delhi, India; ^2^ Clinton Health Access Initiative, New Delhi, India

**Keywords:** anti-oxidant, herbal, immune system, SARS-CoV-2, COVID-19

## Abstract

**Background:**

This review analyzed the magnitude of the COVID-19 pandemic globally and in India and the measures to counter its effect using natural and innate immune booster molecules. The study focuses on two phases: the first focuses on the magnitude, and the second on the effect of antioxidants (natural compounds) on SARS-CoV-2.

**Methods:**

The magnitude of the prevalence, mortality, and comorbidities was acquired from the World Health Organization (WHO) report, media, a report from the Ministry of Health and Family Welfare (MoHFW), newspapers, and the National Centre of Disease Control (NCDC). Research articles from PubMed as well as other sites/journals and databases were accessed to gather literature on the effect of antioxidants.

**Results:**

In the elderly and any chronic diseases, the declined level of antioxidant molecules enhanced the reactive oxygen species, which in turn deprived the immune system.

**Conclusion:**

Innate antioxidant proteins like sirtuin and sestrin play a vital role in enhancing immunity. Herbal products and holistic approaches can also be alternative solutions for everyday life to boost the immune system by improving the redox balance in COVID-19 attack. This review analyzed the counteractive effect of alternative therapy to boost the immune system against the magnitude of the COVID-19 pandemic.

## Introduction

The wave of coronavirus has created panic, phobias, and traumatic stress disorder. The immune defense system of the human body is not strong enough to fight against it. Coronavirus is an enveloped virus with a positive single-stranded RNA (ssRNA) genome and nucleocapsid. It is the largest RNA virus with a range of 26–32 kilobases. The spike glycoproteins are present on the viral envelope that binds to certain receptors of the host cells. They are more critical in terms of involvement of infection by the virus. Severe acute respiratory syndrome coronavirus 2 (SARS-CoV-2) affects the upper respiratory tract and enters the host cells via angiotensin-converting enzyme-2 (ACE-2), through their spike (S) glycoprotein, recognized by the TLR7 present on the endosomes. There are two domains of the S protein: S1 and S2. The S1 domain is a receptor-binding domain, and S2 releases the genetic materials into cells by catalyzing the membrane fusion. It enters the airway epithelium, inhibits the host innate interferon (IFN) immune response, and starts replication due to activation of the nuclear factor kappa B (NF-κβ) transcription pathway (1), thereby initiating the activation of inflammation and secreting massive inflammatory cytokines. This enhanced the accumulation of reactive oxygen species (ROS) and suppressed nuclear factor-like-2 (NRF2), which arbitrates the host antioxidant immune system. It has been reported that activation of NRF2 suppresses the replication of SARS-CoV-2 and the inflammatory response (2). So, in the first phase of coronavirus attack, oxidative stress increases due to the suppression of innate immune response, and in the second phase, acute inflammation starts damaging the respiratory system due to a lack of sufficient antibodies (3). Coronavirus affects prominently older people than younger generations due to the lack of naive T cells (4).

Coronavirus being an RNA virus undergoes continuous mutation leading to alteration in the protein sequence of its spike and within itself. These alterations have led to variations in virus transmission, replication, and severity (5). There are several human coronavirus variants from different demographic locations such as the UK variant (B.1.1.7. lineage), Brazil variant (P.1 lineage), US variant (B.1.429 lineage), and China variant. During the second wave, the most noticeable SARS-CoV-2 variants are called the variants of concern (VOCs) comprised of the UK lineage (N501Y), South African lineage (B.1.351) (K417N, E484K, and N501Y) (6), and Brazil lineage (K417T, E484K, and N501Y) (7). In the closing phase of 2021, a new variant known as the “B.1.1.529” emerged, which showed a high rate of transmissibility due to a large number of mutations in its spike protein. Omicron was classified by the WHO as VOC (8, 9), and since then, there have been reports of subvariants of Omicron which include Omicron BA.1 (B.1.1.529), BA.2, and BA.3 (10). Then, the UK reported the first case of the XE variant, which is a combination of the BA.1 and BA.2 subvariants of Omicron (11).

A new recombinant was identified in France named “Deltacron” (officially known as XD and XF), which is the recombinant of Delta and Omicron variants (10). It is believed that the XE variant is highly contagious when compared with BA.2 and Delta.

In India, “Delta and Delta Plus” variants were reported, which indicates the presence of two mutations, i.e., E484Q, E484K, and L452R (B.1.617 lineage) in the same strain of one deletion (H146 and Y145) and two mutations (E484K and D614G) (12–14). In September 2022, the majority of samples were of the subvariant BA.2.75.2 (15). Though the cases of coronavirus are decreasing, still the emergence of mutants is the major risk factor in the population. Numerous antioxidant plant products have been known since ancient times for their ability to modulate the immune system by immunomodulatory mechanisms, through stimulation of both innate and adaptive humoral and cellular immunity. These mechanisms influence proinflammatory pathways and modulation of the gut microbiome.

This review analyzed the magnitude of the coronavirus disease 2019 (COVID-19) pandemic globally and in India with its prevalence, mortality, and comorbidity scenario and the counteractive effects of antioxidants as an immune booster.

## COVID-19 data—global and India

The first symptom of COVID-19 was identified on 1 December 2019 (16), and since then, it has spread to 231 countries (17, 18). Despite a global effort to contain the disease, it has hamstrung health systems and shaken economies.

Initially, four individuals with “pneumonia of unknown etiology” were reported, all linked to the Huanan (Southern China) Seafood Wholesale Market (19). The disease then rapidly spread across Hubei and nearby provinces.

**Figure 1:**
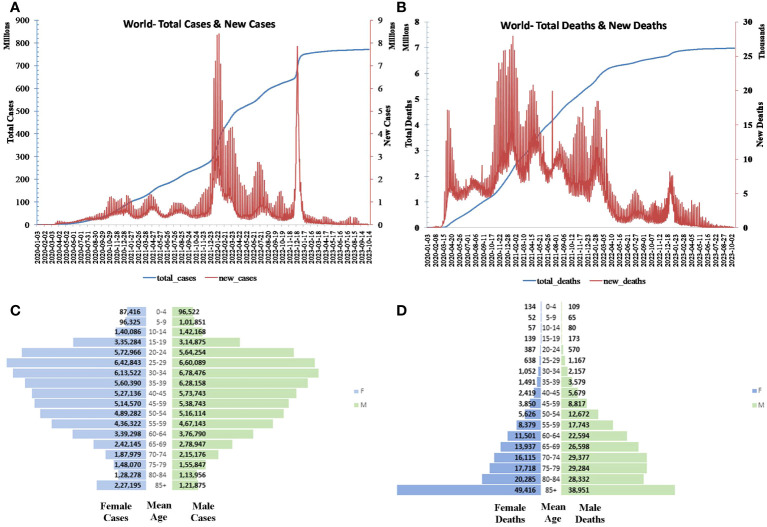
Global **(A)** daily and total cases and **(B)** death progression. Global **(C)** population pyramid cases and **(D)** deaths.

The first case outside China was reported to the WHO on 13 January 2020, in Thailand, which was of a Chinese woman who had traveled from Wuhan, China, to Thailand on 8 January 2020 (20). The disease then spread rapidly in Eastern Asia and Europe. The progress of COVID-19 in each country is mediated by the public health response implemented in that country. The global stats of confirmed cases and deaths are provided in **Figures 1A, B** (21).

As reported by UN Women, COVID-19 predominates in the age group 20–34 among women and 25–39 among men and declines as age and/or exposure increases. Conversely, the mortality increases with age and is particularly lethal for those aged greater than 85 (**Figures 1C, D**) (22).

In India, the first confirmed case of COVID-19 was reported on 30 January 2020 by NIV Pune, of a 20-year-old female who presented after 1 day of symptoms (23). Daily cases peaked in the middle of September, after which there has been a steady decline to June–July levels despite a sustained level of testing by a network of public and private labs. After a steep regression, the number of positive cases began to increase by March 2021 and attained the extent of 4 lakh cases per day and more than 4,000 confirmed death cases. This second wave of the COVID-19 pandemic has been more devastating than the first. This absurd rise resulted from an increased mutation in the RNA genome spreading at a faster pace in every age group. Compared with the first wave, India recorded a two-fold increase in positive cases in the second wave (24, 25). The third COVID-19 wave began with the rise of Omicron cases which was marked as VOC by the WHO.

Among the two Omicron variants, the BA.2 variant accounts for the majority of cases. During this phase, there have been reports of new variants like BA.2.10, BA.2.12, XE, BA.4, BA.5, XBB.1.16, and XBB.1.16.1 (26).

Though COVID-19 patients with Omicron are not adversely affected, people with comorbidities, young children, and the elderly remain at high risk of adverse infection.

The severity of and the mortality due to COVID-19 have been noticed in patients having comorbidities, which is probably due to a lack of immunity caused by ROS imbalance.

## Immune system

The immune system is a massive system with many forces to restrict the entry of foreign bodies and kill them. These forces include many different cells, organs, and proteins that work together to fight against microbes to keep the body healthy.

The innate immune system inherent at birth consists of special immune cells like white blood cells or leukocytes (B cells, T cells, natural killer cells) which fight against harmful substances and germs. The spleen, bone marrow, thymus, skin, lungs, and digestive tracts are the main organs involved in the immune defense system of the body.

The adaptive immune system makes antibodies specifically suitable to fight certain germs. So, antibodies are special proteins that code specific antigens.

Once the microbe breaches the first line of defense, the adaptive immunity gets activated to prevent the spread of the microbe-induced infection. There are specific receptors like Toll-like receptors (TLRs) that recognize the pathogen-associated molecular patterns (PAMPs) present on the pathogen’s surface to prevent entry and further spread by activating the cell-mediated immune response. The infection causes the release of cytokines by the immune cells to communicate and activate B and T cells, thereby preventing the disease’s further spread (27). In particular, viruses have developed mechanisms to evade the immune system and take over the host machinery to cause further infection, and one such virus is coronavirus (CoV) (28).

Antioxidant molecules enhance the humoral immunity of the body during viral infection to maintain the redox balance and thus prevent the replication of the virus.

## Effect of antioxidant molecules on the immune system

The endogenous antioxidant molecules maintain the ROS level by redox balancing, thereby fighting against oxidative stress and maintaining the defense system. ROS is also produced by numerous exogenous factors like exposure to pathogens like viruses and bacteria.

The antioxidant enzymes like superoxide dismutase (SOD), catalase (CAT), glutathione (GSH), and many other antioxidant proteins like sirtuin (SIRT), sestrin (SESN), and forkhead box transcription factor (FOXO) have a critical role in maintaining the level of the antioxidant molecules to balance the immune system. This immune system is also enhanced by supplying antioxidant molecules through diet.

Some non-enzymatic compounds like zinc, vitamin C, vitamin D, vitamin E, flavonoids, curcumin, and selenium prevent the entry of viruses into the body via different mechanisms. Zinc is known to have concentration-dependent effects on ACE-2 activity as it has low affinity-binding sites for zinc (29). Various *in-vitro* and human clinical studies demonstrate the antiviral activity of zinc such as inhibiting coronavirus Rdrp template binding/elongation and reduction in outbreak recurrence in the case of herpes simplex virus in human subjects (30). Zinc prevents viral entry by protecting the cell membrane from non-specific leakage (31). Vitamin C is known to modulate the expression of ACE-2 receptors in human small alveolar epithelial cells and microvascular endothelial cells (32). Vitamin D also downregulates ACE-2 receptor activity and acts as a negative regulator of RAS (33). Vitamin E does not have a direct relation with disrupting viral entry but it helps maintain strong immunity. It increases antibody titers after vaccination for hepatitis B, tetanus, and influenza infection and has been shown to decrease the incidence of pneumonia in elderly men (34). Selenium apart from downregulating the ACE-2 receptor also inhibits viral proteases of SARS-CoV-2 (35). Curcumin inhibits ACE-2 receptor, viral protease, and viral S protein (36, 37). Flavonoids such as epigallocatechin gallate found in green tea interact with proteins on the surface of virions of many viruses and prevent their attachment to the host cell (38). Moreover, flavonoids can bind to the spike protein, helicase, and protease sites on the ACE-2 receptor and inhibit viral entry of coronaviruses (39).

## Effect of antioxidant molecules on SARS-CoV-2

The antioxidant enzyme SOD breaks the toxic oxygen molecules (O_2_^−.^) into hydrogen peroxide (H_2_O_2_), which is responsible for various diseases. It is the first line of defense against ROS and maintains the redox balance. Hydrogen peroxide (H_2_O_2_) is further neutralized to water through enzymatic reactions by CAT or GSH. Coronavirus like other RNA viruses (40) activates oxidative stress. The analog of GSH, such as N-acetylcysteine, has an immunomodulating effect and destructive action on viruses by blocking viral replication (41). COVID-19 infection is more common in the elderly and various age-associated diseased patients, who are already in oxidative stress resulting from an increase in viral replication. Overproduction of cytokines occurs due to viral infections, which can only be reduced by antioxidant molecules. Excessive SOD can control harmful oxygen, and then CAT can regulate cytokine production in leukocytes, thus protecting the alveolar cells and suppressing the replication of the COVID-19 virus (42). The expression of SOD is reported to be reduced due to COVID-19 infection in the lungs of elderly patients (43). COVID-19-positive patients have a high profile of interleukins (IL-6, IL-10, etc.) and tumor necrosis factor-alpha (TNF-α), causing a cytokine storm and suppression of GSH. COVID-19 also alters the GSH level by reducing the function of NRF2, which is involved in GSH upregulation. Studies have shown that extracellular administration of liposomal GSH helps to boost the immune system and protect against oxidative damage (44).

There are three metallic SODs present in the catalytic core: manganese (Mn), copper (Cu), and zinc (Zn) SOD. The increased transcription of Cu/Zn-SOD and Mn-SOD would benefit the immune system to defend against pathogen infection (45). Zinc modulates the entry of viruses (30). Exposure to Zn reduced the activity of human ACE-2, which is the binding receptor of coronavirus (29) and reduces the duration of viral symptoms (46). Zn^2+^, combined with its ionophore pyrithione, has been reported to inhibit the RNA-polymerase activity of coronavirus by inhibiting its replication (47).

Coronavirus infection reduces nicotinamide adenine dinucleotide (NAD^+^), a cofactor involved in the oxidation–reduction reaction, disrupting mitochondrial activity and deacetylase activity of SIRT (48). The antioxidant protein SIRT is a NAD^+^-dependent deacetylase protein. It triggers the activation of the SOD enzyme (49). SIRTs are a family of seven proteins (SIRT1–7). **Table 1** illustrates the various cellular processes in which different human sirtuins are implicated. Among them, SIRT1 and SIRT3 are mainly involved in controlling ROS production. In a culture system of H_2_O_2_-treated R28 cells, SIRT1 and SIRT3 levels were found to be decreased, and the ROS level was elevated. However, both SIRT levels increased with the decrease of ROS level after treatment with the antioxidant glucagon-like peptide 1 analog, exendin-4 (EX4) (56). Consequently, the levels of mRNA and SOD were found to be elevated. SIRT deacetylates many proteins like p53 and NF-κβ. SIRT1 deacetylase p65 and reduced NF-κβ transcriptional activity on endothelial cells. NF-κβ plays a central role in inflammatory cytokine stimulation and lymphocyte activation. Thus, SIRT1 has many effects on immunity (57). In a cytokine storm syndrome due to COVID-19 infection, all the proinflammatory molecules like TNF-α, IFN-g, IL-6, and IL-1 express abundantly, affecting the innate immune system (58). SIRT1 also activates T cells by deacetylating FOXO via modulation of SOD and CAT (51, 52). Silencing SIRT1 in T cells of mice increases inflammatory molecules, and activation of SIRT1 by resveratrol and SRT172 reduces ROS and NF-κβ (55). Many SIRT activators have been found to boost immunity by regulating ROS production. Plant products like *Syzygium aromaticum (* 58) were reported to enhance SIRT1 level and thus increased the antioxidant enzymes SOD, CAT, GSH, etc. In aging, the level of SIRTs is found to be downregulated (59); hence, the severity rate of aged COVID-19 patients is higher. It has been found that viruses like HCMV, HSV-1, and Ad5 and the influenza virus H1N1 are increased by siRNA-mediated knockdown of individual human SIRTs in cultured cells. The viruses also produce more through treatment with the SIRT1 inhibitor and are reduced by its activator (53). The exact mechanism of action of SIRT on preventing virus replication is not known. As SIRT is a NAD^+^-dependent deacetylated enzyme, it influences metabolic functions and has various roles in cellular functions. It inhibits fatty acid synthesis and glycolysis by deacetylating the concerned proteins, which may prevent viral growth. Considering the diverse role of SIRT mainly on antioxidant properties that boost the immune system, it can be revealed that SIRT may be a potential target for preventing COVID-19 infections.

**Table 1:** Human sirtuins and their functions.

Human sirtuin	Functions	References
**SIRT1**	Deacetylates p65 and NF-κβ, thus modulating inflammatory cytokine stimulation and lymphocyte activation Deacetylates FOXO and activates T cells, upregulates ROS scavengers: SOD2 and catalase Prevents viral replication in HCMV, HSV-1, Ad5, and influenza virus H1N1 Inhibits ADAM-17 leading to inhibition of TNF-α and IL-6	(50) (49, 51, 52) (53) (48)
**SIRT2**	Deacetylates MYC, FOXO3A, tubulin, G6PD, EIF5A Regulates apoptosis via p53 deacetylation Regulates cell cycle progression at G2/M and metaphase to anaphase checkpoint	(54) (54) (54)
**SIRT3**	Deacetylates FOXO3A Adapts the mitochondria to starving or caloric restriction Increases the activity of the enzymes acetyl coenzyme A (CoA) synthetase 2 (AceCS2), long-chain acyl-CoA dehydrogenase (LCAD), and ornithine transcarbamylase (OTC)	(55) (55) (55)
**SIRT4**	ADP ribosylation	(53)
**SIRT5**	Desuccinylation and demalonylation	(53)
**SIRT6**	ADP ribosylation Hydrolysis of long-chain fatty acyl lysine Involved in cellular homeostasis and DNA repair	(53) (53) (54)
**SIRT7**	Deacetylates Hif-1α/2α	(54)

Sestrin is another potential antioxidant protein. It is a stress-inducible protein and protects the cells by regulating oxidative stress, endoplasmic reticulum stress, autophagy, metabolism, and inflammation. It has a preventive function on immune cells by activating 5′ adenosine monophosphate-activated protein kinase (AMPK) by inhibiting the mammalian target of rapamycin complex 1 (mTORC1) due to its association with the autophagy-related gene and suppressing activation of the c-Jun N-terminal kinase (JNK) pathway (60, 61). ROS accumulation and activation of mTORC1 are more in aging; hence, the stress-inducible protein SESN increases more to overcome the crisis in aging and age-associated disease. In COVID-19 patients, the spike glycoprotein of SARS-CoV-2 binds with the ACE-2 receptor of the host, which is phosphorylated by AMPK, thus altering the structure to prevent the entry and replication of the virus. The SESN protein directly activates AMPK; hence, the SESN protein might have an important role in the immune system to protect against virus replication. COVID-19 infection created inflammation in the terminal airway, alveoli, and lung mesenchyme and accumulated ROS. During this oxidative stress, the expression of SESN increases for autophagy induction through activation of AMPK and ROS clearance. SESN also upregulates NRF2 signaling by promoting p62-dependent autophagic degradation, which overexpresses other antioxidant genes (62). The activators for NRF2—sulforaphane and bardoxolone methyl—are already in clinical trials in COVID-19 (63). It can be proposed that SESN can also serve as one of the potential target molecules for COVID-19 prevention.

FOXO is another important antioxidant protein that maintains ROS formation through autophagy by regulation of manganese superoxide dismutase (MnSOD) and CAT (64).

Vitamin C has an essential role in maintaining the various immune systems. It increases the production of natural killer cells (NK cells) and B and T cells to fight against viruses. It has an important homeostatic role as an antioxidant by maintaining the ROS level and inflammation by suppressing NF-κβ activation. It has been reported that in the critical phase of COVID-19, vitamin C plays a serious role in downregulating the cytokine storm, thus supporting tissue repair and improving immune responses against infections. It also stimulates the formation of antibodies (65).

Vitamin D secretes various antiviral peptides to increase innate immunity during viral infection, thus playing an important role in immunomodulation (66, 67). It decreases cytokine storm by inhibiting T-helper cell type 1 responses and also stimulates the induction of T cells, thereby regulating the adaptive immunity (68, 69). Many patients with acute respiratory disease are found to have vitamin D deficiency. Many studies on the recent pandemic reported the deficiency of vitamin D levels in COVID-19 patients (70–75). It has been observed that delivering vitamin D improves the severity and mortality of COVID-19 patients (72–76).

Vitamin E increases NK cells in the immune system and neutralizes ROS. B cells and T cells that fight against viral infections are found to lose their immunity function due to vitamin E deficiency. Though very little is known about the effect of vitamin E on COVID-19 patients, it is advised that COVID-19 patients take vitamin E to increase their immunity.

Many studies have highlighted the effectiveness of antioxidant molecules in boosting the immune system. In a study, critically ill patients with COVID-19 were supplemented with oral selenium and zinc, which resulted in the elevation of selenium and selenoprotein P levels to the normal range (77). This intervention led to a significant decrease in CRP, PCT, IL-6, IL-1β, and IL-10 along with an increase in CD8^+^ T cells, NK cells, and total IgG levels in the patients. In an *in-vitro* study, preincubating immune cells from peripheral blood mononuclear cells (PBMCs) of donors with a history of COVID-19 and uninfected donors with tempol, a novel antioxidant, led to a decrease in different T-cell and APC-derived cytokines (78). In an immunosuppressive mouse model, a solubilized curcuminoid complex was found to lower the levels of neutrophils, dendritic cells, natural killer cells, CD4^+^ T cells, and CD8^+^ T cells in the spleen (79).

## Plausible therapy for protection of COVID-19 targeting antioxidant pathways

The actual cause, pathogenesis, and therapy of COVID-19 are not fully known till now. A lot of studies are going on after observation of the symptoms of COVID-19 patients. The symptoms are varied, and many are asymptomatic. So, it is difficult to define the pathogenesis clearly. Surely, the immunity process in the body is not sufficient to fight against the virus, especially in elderly individuals. The coronavirus mainly infects and creates a worse situation in the elderly. Therefore, it is necessary to boost the immunity system of the elderly whose immune system is already weak.

This virus causes acute respiratory distress syndrome (ARDS) of the upper respiratory tract. The immune system needs more strength and support from an exogenous source as the upper respiratory tract is considered an external part of the body.

The antioxidant components, which play a critical role against the virus, need to be supplied more to boost the immunity to fight against the virus. The lack of endogenous antioxidant products impairs the immune system during coronavirus infection. So, the supply of exogenous antioxidant products may enhance the immune system to fight against viruses. There are many natural products and specifically designed synthetic products that can activate various antioxidant proteins and molecules, to be used in combination as a therapy for the prevention or protection from the virus. Flavonoids, quercetin, catechin, herbacetin, rhoifolin, pectolinarin, myricetin, and scutellarein are potential antioxidant compounds that have antiviral properties as shown in *in-vitro* studies by inhibiting the replication of COVID-19 (80–83). *Syzygium aromaticum* (49, 58), resveratrol (84), the synthetic peptide CWR (85), and the compound like SRT (86) activator of SIRT were found to enhance the secretion of SOD, CAT, and GSH by scavenging ROS. *Syzygium aromaticum (* 87) also regulates the level of SENS to maintain oxidative stress. Ashwagandha (*Withania somnifera*) could inhibit the virus’ entry by blocking the host enzyme transmembrane protease serine 2 (TMPRSS2) (88). Consumption of ashwagandha as a supplement could reduce COVID-19 chances, as the virus fusion with ACE-2 is inhibited due to blocked TMPRSS2 (89). Studies have shown that curcumin inhibits ACE-2 receptor, viral protease, and viral S protein, thus preventing COVID-19 infection (36). Zn can be used as a supplement for COVID-19 prevention as it helps in reducing the occurrence of respiratory infection due to its antiviral and anti-inflammatory properties by inhibiting TNF-α (90).

*Azadirachta indica*, commonly known as neem, is known to exhibit certain protective effects from various diseases such as in metastasis. It prevents cancer cells from cytoadhering and inhibits HIV from penetrating the target T lymphocytes. Moreover, it hampers the spike glycoprotein of HIV from binding CD4^+^ T cells and hinders malaria-parasitized red blood cells (pRBCs) from adhering to the vascular endothelium (VE). In the case of COVID-19, since viral adherence to VE can cause multiple organ distress syndrome, neem can be explored to arrest VE adherence and prevent extreme morbidity and mortality. It may bind to VE cells and block the spike glycoprotein of coronavirus from interacting with VEC ACE-2 (91).

A phytochemical called lycopene, possessing a multitude of antioxidant-based therapeutic properties, may boost the physiological response in resistance to COVID-19 infection (92). Lycopene prevents macrophages from producing proinflammatory cytokines and chemokines, which reduces inflammation in several organs (93, 94). The effects of 13 Bulgarian medicinal plant extracts on the MCR-5 cell line transfected with the human coronavirus 229E strain were studied by Ilieva N. et al. (95) In their study, plant extracts of *T. vulgaris*, *M. chamomilla*, *A. sativum*, and *P. reptans*, among others, were portrayed to have prominent anti-coronavirus activity. Most of the plants suppressed extracellular virions and showed potent redox-modulating effects. Another such study published in February 2022 by Lizdany Flórez-Álvarez et al. (96) studied the antiviral activity of Colombian plant extracts on COVID-19. They transfected Vero E6 cells with SARS-CoV-2 viral stock and treated them with plant extracts. They found two plant extracts from *P. tuberculatum* and *G. sepium* to effectively inhibit SARS-CoV-2.

In some *in-silico* studies, plant extracts have been shown to be active against coronaviruses. *Hibiscus sabdariffa* L., commonly called the roselle flower, has three major active ingredients, namely, anthocyanins, ascorbic acid, and tartaric acid. The *in-silico* interaction of these active ingredients with the ACE-2 spike and other inflammatory target proteins was studied by Ramadhani et al. (97) Anthocyanin had the best binding affinity with all the target proteins. For the ACE-2 spike and IL-10, the binding affinity with anthocyanin was −7.5 kcal/mol and −6.5 kcal/mol, respectively. In a paper published in *Phytomedicine* by Mukherjee PK et al. (98), approximately 95 medicinal plants have been listed that were found to have anti-inflammatory or antioxidant activities reported between 2003 and 2021. They proposed to explore the use of these medicinal plants as therapy against COVID-19. **Table 2** depicts various natural products along with their immunological effects on COVID-19.

**Table 2:** Natural products and their immunological effects on COVID-19.

Natural product	Effect	Targets	References
**Flavonoids, quercetin, catechin, herbacetin, rhoifolin, pectolinarin, myricetin, scutellarein**	Inhibits the replication of SARS-CoV-2	SARS-CoV 3CLpro	(67–70)
***Syzygium aromaticum*, resveratrol**	Increases SOD, CAT, GTH	SIRT1	(40, 47, 74)
***Withania somnifera* (ashwagandha)**	Inhibits the entry of virus by blocking the host enzyme TMPRSS2	TMPRSS2	(75, 76)
**Curcumin**	Inhibits ACE-2 receptor, viral protease, and viral S protein	ACE-2 receptor, viral protease, viral S protein	(77)
***Azadirachta indica* (neem)**	Prevents malarial pRBCs from adhering to the vascular endothelium, inhibits HIV from penetrating the target T lymphocytes	Vascular endothelium	(99)
**Lycopene**	Boosts physiological response to COVID-19 Prevents macrophages from producing proinflammatory cytokines and chemokines	Nitric oxide, nitric oxide synthase, NF-κβ, TNF-α	(79) (80, 81)
***T. vulgaris*, *M. chamomilla*, *A. sativum*, *P. reptans***	Anti-coronavirus activity, suppresses extracellular virions, redox modulation	ROS, human coronavirus virions	(82)
***P. tuberculatum*, *G. sepium***	Inhibits SARS-CoV-2	–	(83)
***Hibiscus sabdariffa* L. anthocyanin**	Binds ACE-2 spike and IL-10	ACE-2 spike, IL-10	(84)

**Figure 2:**
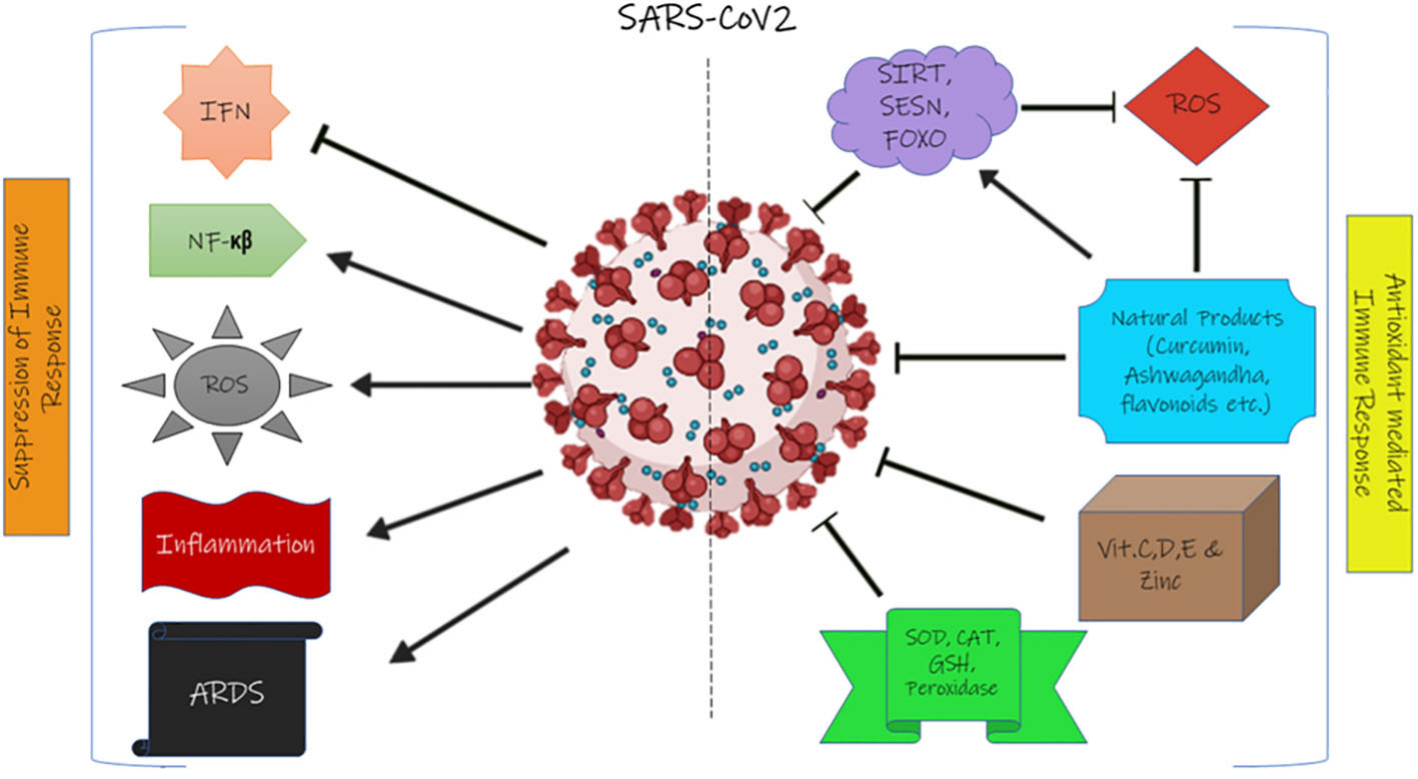
The effect of SARS-CoV-2 on the immune system and the effect of the antioxidant system on coronavirus. IFN, interferon; ROS, reactive oxygen species; ARDS, acute respiratory distress syndrome; SIRT, sirtuin; SESN, sestrin; FOXO, forkhead box transcription factor; SOD, superoxide dismutase; CAT, catalase; GSH, glutathione.

**Figure 2** illustrates the suppression of the immune system by SARS-CoV-2 and the effect of natural antioxidant molecules on it.

There are some reported studies that demonstrate the practical applications of antioxidant molecules in SARS-CoV-2 infection. In one such case, ambulatory COVID-19 patients, in addition to standard therapy, were supplemented with a mixture of combined metabolic activators (CAMs) including glutathione and NAD^+^ precursors or placebo to investigate the effect of CAMs on symptom-free recovery time (100). According to the findings, the CMA group’s duration to full recovery was considerably less than that of the placebo group in both phase 2 and 3 trials. The CMA group also portrayed considerably better levels of plasma proteins and metabolites linked to inflammation and antioxidant metabolism than the placebo group.

Another randomized controlled trial assessed the potential of quercetin to impede the progression of SARS-CoV-2 and lower relevant inflammatory markers (101). In the control group, only antiviral drugs were administered, whereas the intervention group received both antivirals and 1,000 mg of quercetin every day for 7 days. The intervention group was found to have lower levels of inflammatory molecules, namely, alkaline phosphatase, quantitative C-reactive protein, and lactate dehydrogenase, in the serum.

It is a great challenge for the speedy development of vaccines with full clinical efficacy and drugs against COVID-19 in this pandemic situation. COVID-19 vaccination drives are in full swing all over the world.

## Vaccination

The development of vaccines for COVID-19 commenced in early 2020. Since then, several vaccine candidates have been found to be efficacious in reducing the severity of the disease as well as in preventing infection in clinical trials.

Vaccine breakthrough infections, as mentioned by the CDC, are COVID-19-positive cases arising even after being immunized with a primary series of vaccination courses or even with additional booster doses after the primary series. Though vaccinated individuals are likely to experience milder symptoms compared with the unvaccinated ones, they can still pose as disease carriers for others. During the higher number of cases being reported in a population of individuals, the viral load in that population is significantly high. Higher viral load causes more number of vaccine breakthrough infections, despite having high vaccination rates (102). However, the death rates seemed to decrease with a higher series of vaccination (103).

One limiting feature of vaccines is their stability. Vaccine stability is the capability of a vaccine to retain its specific properties till the end of its shelf life. This attribute is mainly related to mRNA vaccines which show instability related to the vaccine’s critical quality attributes (*in-vitro* stability) or to mRNA’s intrinsic features (*in-vivo* stability). Therefore, an mRNA vaccine requires an extremely low temperature and specific environmental conditions so as to retain its stability. To overcome mRNA instability, various techniques are employed. Retaining mRNA integrity, altering mRNA fragment length, optimizing mRNA sequence, modifying mRNA, and using circRNA are found to be useful in increasing the shelf life of the vaccine. In addition, suitable excipients, appropriate lipid nanoparticle (LNP) delivery systems, and following strict manufacturing conditions such as pH/temperature maintenance and lyophilization are also crucial to maintain vaccine stability (104).

Vaccine efficacy is also limited by the mutability of the virus. It has been previously found that mutations induced in the spike protein are chiefly responsible for dampening the effects of vaccines which remain effective for various strains but are most effective for the original strain against which they were made. Thus, it causes a need for booster doses (105).

A study by Julián Andrés Mateus Rodriguez et al. published in December 2021 quizzed whether the nutritional supplement of ABB C1^®^, which is a β-glucan complex and a consortium of *S. cerevisiae* enriched with selenium and zinc, could boost the immune response of participants who had taken influenza vaccine (*n* = 34) or Pfizer-BioNTech’s COVID-19 mRNA vaccine (*n* = 38). The participants were divided into the ABB C1^®^ group and the placebo group and received supplementation the next day after getting the vaccine. The study found that, after the second dose of the COVID-19 vaccine, the mean levels of CD4^+^ T cells increased in the ABB C1^®^ group, whereas there was a decrease of CD4^+^ T cells in the placebo group. For the influenza vaccine, although the mean levels of CD4^+^ T cells increased in both groups, the magnitude of the increase was higher in the ABB C1^®^ group (106).

Thus, the data suggest that vaccination does seem to have an effect, as shown by the decrease in the death rate, but it is not enough to fully combat the COVID-19 pandemic. Adjuvant precautionary therapy with plant extracts containing naturally active ingredients should be considered a plausible solution, in addition to vaccination.

## Discussion

The SARS-CoV-2 pandemic has resulted in the loss of thousands of lives and has led to various other side effects of post-infection. Global and local data depicted the enhanced spread and mortality caused by this novel virus. Comorbidity data showed that patients with diabetes, hypertension, and chronic obstructive pulmonary disease (COPD) are more vulnerable to this infection. An increase in ROS level resulted in the host antioxidant system’s impairment, causing DNA damage and mitochondrial dysfunction. Though global vaccination is the ultimatum for eradicating the COVID-19 pandemic, achieving this goal is a lengthy procedure.

The vaccination drill presently rolling across the world might prove effective, but clinical solid evidence is yet to be established. In the second wave in India, those who have comorbidities at a young age are at a high risk. The virus is becoming more infectious and some mutations escape the immune response, which infects even after vaccination. After the regression of the second wave, the fear of a possible third wave is high. It is believed that if the third wave hits countries like India, the younger population, i.e., the age group below 18, would be more prone to infection due to lack of vaccination. Though the majority of vaccinated people are having mild symptoms, still the risk of severity persists. The alternative holistic therapeutic intervention to boost immunity for the prevention of coronavirus may be the successful eradication of the disease. All these variations and variabilities lead to dependence on natural and innate immune molecules such as vitamin D, vitamin E, sirtuins, sestrins, FOXO, and catalase. Augmentation of the above innate molecules enhances the immune system’s ability to counter the redox imbalance and has no harm or side effects on one’s health. Thus, supplementation of natural products along with the administration of vaccine could boost immunity to fight against these VOCs. Though antioxidant supplementation with natural products looks encouraging, there are a few limitations to be overcome. Natural products alone have low solubility, rapid blood clearance, potential toxicity to unwanted sites, and low bioavailability and, thus, require a good lipophilic carrier such as liposome formulations for adequate drug delivery in clinical settings (107). Moreover, allergic dermatitis to natural products such as neem may also be an associated risk for certain individuals (99). Despite these limitations, the therapeutic potential of natural products can be explored as drug delivery research using liposomes is promising, and monitoring for allergic reactions is feasible (107). The review seeks to provide a strong message to use activators of SIRT, SESN, medicinal plants, vitamins, and metals with high antioxidant properties to boost the immune system by controlling the autophagy and maintaining the antioxidant enzymes as a conventional adjuvant therapy against the complex pathophysiology of COVID-19 infections for long-term care.

## Author contributions

SD: concept of review, collected different data, wrote the manuscript, AD: collected all the COVID-19 data both global and India, prepared the figures and table, PK and KR: wrote many parts of the second phase of the manuscript, collected information about it and prepared the references and edited the manuscript. All authors contributed to the article and approved the submitted version.
